# Trueness of 12 intraoral scanners in the full-arch implant impression: a comparative in vitro study

**DOI:** 10.1186/s12903-020-01254-9

**Published:** 2020-09-22

**Authors:** Francesco Guido Mangano, Oleg Admakin, Matteo Bonacina, Henriette Lerner, Vygandas Rutkunas, Carlo Mangano

**Affiliations:** 1 Department of Prevention and Communal Dentistry, Sechenov First State Medical University, 119992 Moscow, Russia; 2 Ars and Technology, Sotto il Monte Giovanni XXIII, 24039 Bergamo, Italy; 3 Academic Teaching and Research Institution of Johann Wolfgang Goethe University, 60323 Frankfurt am Main, Germany; 4 Department of Prosthodontics, Institute of Odontology, Faculty of Medicine, Vilnius University, LT-01513 Vilnius, Lithuania; 5 Department of Dental Sciences, Vita and Salute University San Raffaele, 20132 Milan, Italy

**Keywords:** Intraoral scanner, Full-arch implant impression, Scanbody, Trueness, Comparative study

## Abstract

**Background:**

The literature has not yet validated the use of intraoral scanners (IOSs) for full-arch (FA) implant impression. Hence, the aim of this in vitro study was to assess and compare the trueness of 12 different IOSs in FA implant impression.

**Methods:**

A stone-cast model of a totally edentulous maxilla with 6 implant analogues and scanbodies (SBs) was scanned with a desktop scanner (Freedom UHD®) to capture a reference model (RM), and with 12 IOSs (ITERO ELEMENTS 5D®; PRIMESCAN® and OMNICAM®; CS 3700® and CS 3600®; TRIOS3®; i-500®; EMERALD S® and EMERALD®; VIRTUO VIVO® and DWIO®; RUNEYES QUICKSCAN®). Ten scans were taken using each IOS, and each was compared to the RM, to evaluate trueness. A mesh/mesh method and a nurbs/nurbs method were used to evaluate the overall trueness of the scans; linear and cross distances between the SBs were used to evaluate the local trueness of the scans. The analysis was performed using reverse engineering software (Studio®, Geomagics; Magics®, Materialise). A statistical evaluation was performed.

**Results:**

With the mesh/mesh method, the best results were obtained by CS 3700® (mean error 30.4 μm) followed by ITERO ELEMENTS 5D® (31.4 μm), i-500® (32.2 μm), TRIOS 3® (36.4 μm), CS 3600® (36.5 μm), PRIMESCAN® (38.4 μm), VIRTUO VIVO® (43.8 μm), RUNEYES® (44.4 μm), EMERALD S® (52.9 μm), EMERALD® (76.1 μm), OMNICAM® (79.6 μm) and DWIO® (98.4 μm). With the nurbs/nurbs method, the best results were obtained by ITERO ELEMENTS 5D® (mean error 16.1 μm), followed by PRIMESCAN® (19.3 μm), TRIOS 3® (20.2 μm), i-500® (20.8 μm), CS 3700® (21.9 μm), CS 3600® (24.4 μm), VIRTUO VIVO® (32.0 μm), RUNEYES® (33.9 μm), EMERALD S® (36.8 μm), OMNICAM® (47.0 μm), EMERALD® (51.9 μm) and DWIO® (69.9 μm). Statistically significant differences were found between the IOSs. Linear and cross distances between the SBs (local trueness analysis) confirmed the data that emerged from the overall trueness evaluation.

**Conclusions:**

Different levels of trueness were found among the IOSs evaluated in this study. Further studies are needed to confirm these results.

## Background

Intraoral scanners (IOSs) are changing the world of implant prosthodontics [1, 2]. IOSs use structured light and/or laser to capture sequential images of the patient’s dental arches, allowing three-dimensional (3D) reconstruction of their surface using powerful reconstruction software. These software applications generate point clouds that are triangulated to give surface reconstructions (meshes), i.e. virtual models of the patient’s dental arches [2, 3].

IOS optical impressions allow the dentist to capture virtual models of the patient’s dental arches, with no conventional impression using trays and materials (which have always been unwelcome to patients) and without having to pour any plaster cast, saving time and space [2, 4, 5]. The clinical procedure is technically simplified, and the virtual models can be immediately sent to the laboratory as standard tessellation language (STL) files, without disinfection or shipping costs [2, 5, 6]. The optical impressions improve the communication with the dental laboratory, which becomes more efficient, and represents the gateway to the world of computer-aided design and manufacturing (CAD/CAM) [5, 6].

To date, in fixed implant prosthodontics, the scientific literature has validated the use of IOSs for capturing optical impressions for the design and manufacture of short-span restorations such as single crowns (SCs) [7–10] and partial prostheses (PPs) [11–13]. However, in the case of long-span restorations, and in particular for full arches (FAs), IOSs do not yet seem to be sufficiently accurate, as reported by several studies [14, 15] and reviews of the literature [16, 17].

In metrics, accuracy is “the closeness of agreement between a measured quantity value and a true quantity value of a measurand” (JCGM 200:2012; ISO 5725–1, 1994) [2, 4, 13], and when it comes to IOSs, it is the combination of trueness and precision. Trueness is the most important factor, defined as “the closeness of agreement between the arithmetic mean of a large number of test results and the true or accepted reference value”. Trueness expresses how much the average of a series of measurements approaches reality; a measurement is truer the closer it is to the actual value of the object. To evaluate the trueness of a measurement requires a reference: in the case of dental models, this is an acquisition made with a machine with certified accuracy (possibly ≤5 μm), such as a coordinate measuring machine (CMM), or an industrial optical or desktop scanner [2, 4, 13]. Specifically, the acquisitions obtained with IOSs must be compared with those obtained with one of these reference machines to be mathematically validated. Precision is “the closeness of agreement between measured quantity values obtained by replicate measurements on the same objects under specified conditions”: it refers to the closeness of agreement and deviations between test results. To evaluate precision does not require a reference: it is sufficient to compare the measurements made with the same IOS and evaluate the deviations between them [2, 4, 14].

Because it is not possible to use machines with certified accuracy such as CMMs, articulated arms or industrial scanners in the patient’s mouth, and having a certified reference file is not possible, measuring the trueness of optical impressions with IOS in vivo is difficult [2, 4, 18]. Some authors have recently tried to introduce indexes [19, 20] or geometric shapes with known dimensions (custom measuring aids) [21, 22] in the mouth to evaluate the distortions affecting the optical impression in vivo, but the vast majority of studies of the trueness of IOSs have been made in vitro on plaster models [14, 15, 23–26].

Among these in vitro studies, many have used a mesh/mesh method, directly superimposing the meshes (virtual models) derived from intraoral scanning with different IOSs onto a reference mesh obtained with a certified industrial or desktop scanner [14, 15, 23, 24]. Although this approach is intuitive and immediate, and provides reliable information about the overall trueness of a scan, it has limitations. First, it uses meshes that are surface reconstructions and therefore geometric approximations of the scanned model, on which it is impossible to perform reliable distance calculations between the different scanbodies (SBs), i.e. the digital transfers of the implant position. In addition, this approach does not really replicate what happens in the early stages of prosthetic CAD modelling. In implant prosthodontics, the first CAD step involves replacing the mesh (which is a surface reconstruction and therefore a geometric approximation) of the SB with the corresponding SB library file, available in the manufacturer’s implant library [1, 25–27]. This library file, although saved in the same STL format, is not the result of a 3D acquisition (and therefore a surface reconstruction with geometric approximation, such as a mesh): it is a geometrically perfect file or a non-uniform rational b-spline (nurbs) file, originally designed in CAD, and linked to all the other components of the implant library (bonding bases of different height and diameter) on which the dental technician models the individual abutment or the restoration directly [25–27]. For this reason, investigating the accuracy of an intraoral scan after replacing each of the SBs in the mesh with the corresponding library file, and then superimposing two nurbs files (the position of the SBs in the space obtained with the reference scanner and with the IOSs, respectively), may be important to obtain more reliable information on the overall trueness and to be able to calculate the exact distances between the SBs with 3D calculation software, after having automatically identified their centroids. This approach requires substantial work and hundreds of superimpositions but is probably the most suitable to be able to identify the overall and local trueness (distances between the SBs) of an IOS, considering that the distances between the SBs cannot be properly calculated on a mesh [26–28].

The purpose of this in vitro study was therefore to assess and compare the overall trueness of 12 different IOSs, using two different investigation methods (mesh/mesh and nurbs/nurbs superimposition), and to calculate the exact distances between the different SBs (linear distances between the SBs during the progression of the scan and cross distances, i.e., distances between SBs with different positions in the arch).

## Methods

### Study design

The present in vitro study was designed to assess and compare the trueness of 12 different IOSs (ITERO ELEMENTS 5D®, Align Technologies, San José, CA, USA; PRIMESCAN® and OMNICAM®, Dentsply Sirona, York, PA, USA; CS 3700® and CS 3600®, Carestream Dental, Atlanta, GA, USA; TRIOS3®, 3-Shape, Copenhagen, Denmark; i-500®, Medit, Seoul, South Korea; EMERALD S® and EMERALD®, Planmeca, Helsinki, Finland; VIRTUO VIVO® and DWIO®, Dentalwings, Montreal, Canada; RUNEYES QUICKSCAN®, Runeyes Medical Instruments, Ningbo, Zhejiang, China) in FA implant impression.

**Fig. 1:**
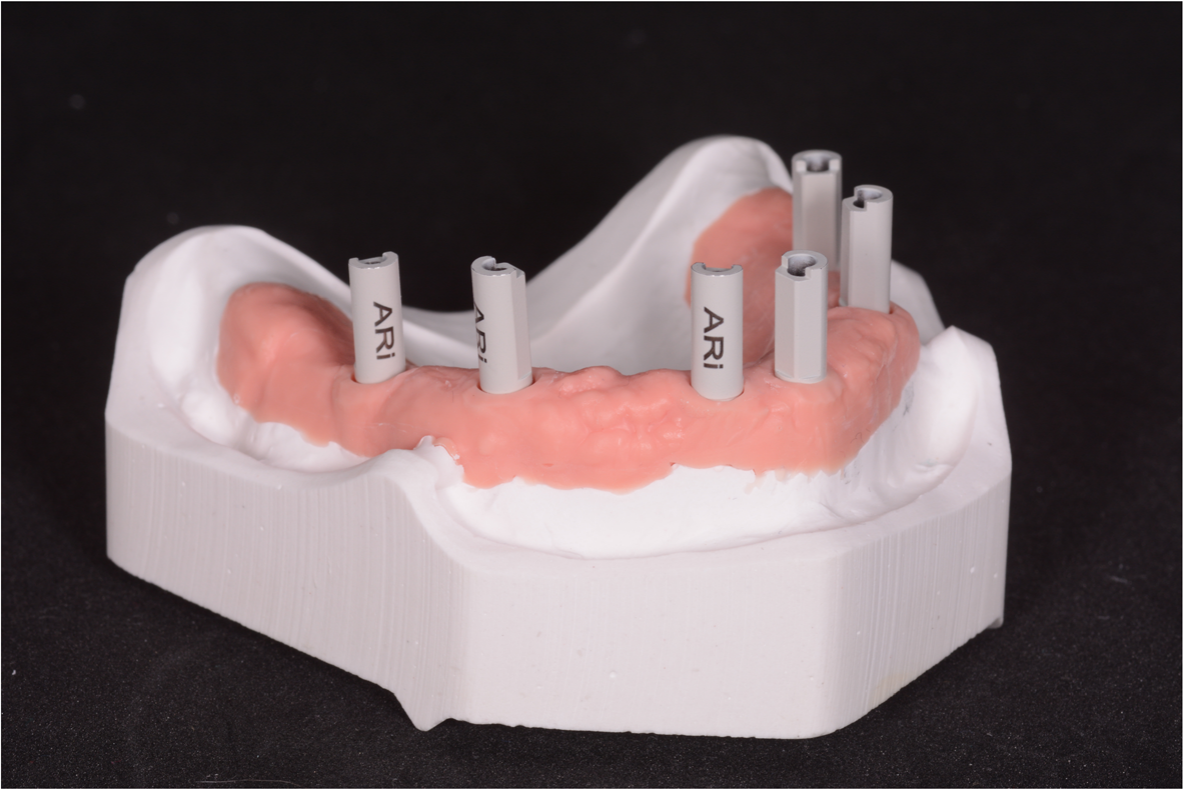
In this in vitro study, a type IV gypsum model was used. This model represented a totally edentulous maxilla with 6 implant analogues in positions #11, #14, #16, #21, #24 and #26 (right and left central incisors, first premolars and first molars) and high-precision non-reflective polyether-ether-ketone (PEEK) SBs (Megagen®, Daegu, South Korea) screwed on

This study used a type IV gypsum model representing a totally edentulous maxilla with 6 implant analogues in positions #11, #14, #16, #21, #24 and #26 (right and left central incisors, first premolars and first molars) and high-precision non-reflective polyether-ether-ketone (PEEK) SBs (Megagen®, Daegu, South Korea) screwed on (Fig. 1). The SBs were named by convention S1 (#26), S2 (#24), S3 (#21), S4 (#11), S5 (#14) and S6 (#16). The model, which had been also used in a previous study [14], presented pink gum in the areas of the implant analogues and simulated the situation of an implant-supported fixed FA prosthesis.

The gypsum model was first scanned with a powerful desktop scanner (Freedom UHD®, Dof Inc., Seoul, Korea), to acquire reference virtual models. This desktop scanner uses a white light-emitting diode and has two 5.0 megapixel cameras. It works under patented stable scan stage technology, which allows the cameras and lights to move and rotate above and around the model, which remains stationary. This allows rapid and effective capture of all details of the model in a few steps and in less than 1 min, generating virtual models in STL immediately usable by any CAD. The scanner has dimensions of 330 × 495 × 430 mm and a weight of 15 kg, is powered at 110–240 V and 50–60 Hz, and works with Windows operating systems 7, 8, and 10 (64-bit). Three virtual models were captured with this desktop scanner and saved in a dedicated folder, labelled with the scanner name. Then, the quality of these meshes was investigated with reverse engineering software (Studio®, Geomagics, Morrisville, NC, USA), and always within the same software, the models were cut and trimmed in order to isolate the SBs and to eliminate the pink gingiva area. Once cut and made uniform, these meshes were superimposed for the validation of the superimposition method and for the choice of the reference model (RM) to be used in the study, as previously described [29]. The RM was saved in a specific folder, ready for use.

After the preparation of the desktop RM was completed, a single operator with over 10 years of experience with intraoral scanning (FGM) began to capture the scans with each of the IOSs in the study. All scans were taken in a 2-month period (January–February 2020) with the latest acquisition software available for each IOS at that time. The main characteristics of the IOSs used in this study are summarised in Table 1. In all, the operator captured 10 scans for each IOS. The scans had to include the entire area of the pink gum and the 6 different SBs in full. To minimise the potential effects of fatigue, the sequence of scan capture with the different IOSs was randomised. The scans were spaced from each other by a rest period of 5 min for the operator. In all cases, the operator started from the posterior sectors (right or left) and proceeded along the arch with a zig-zag technique. As reported in previous studies [14, 27], this technique provided for a slow and constant advancement of the scan along the arch, starting from the buccal area and then moving occlusally and palatally, and returning to the buccal area: an arch was described above the pink gum and implant SBs. All scans were captured in the same environmental conditions, in a room moderately lit by sunlight with a temperature of 21 °C, humidity of 45% and air pressure of 750 ± 5 mm. Ten virtual models were captured for each IOS, for a total of 120 STL files. These files were saved in dedicated folders, labelled with the IOS name and progressively numbered from 1 to 10.

**Table 1:** Features of the IOSs investigated in this study

Name	Manufacturer	Acquisition technology	Output files
ITERO ELEMENTS 5D®	Align Technologies, San Josè, CA, USA	Parallel Confocal Microscopy	3ds (proprietary format); ply and stl (open formats)
PRIMESCAN®	Dentsply Sirona, York, PN, USA	High-resolution Sensors and Shortwave Light with Optical High Frequency Contrast Analysis for Dynamic Deep Scan (20 mm)	dxd (proprietary format) with possibility to export .stl files (open format) with Cerec Connect®
OMNICAM®		Optical Triangulation and Confocal Microscopy	cs3, sdt, cdt, idt (proprietary format) with possibility to export .stl files (open format) with Cerec Connect®
CS 3700®	Carestream Dental, Atlanta, GA, USA	Active Triangulation with Smart-shade Matching via Bidirectional Reflectance Distribution Function	dcm (proprietary format); ply and stl (open formats)
CS 3600®		LED light scanner -Active Speed 3D Video	csz (proprietary format), ply and stl (open formats)
TRIOS3®	3-Shape, Copenhagen, Denmark	Confocal Microscopy and Ultrafast Optical Scanning	dcm (proprietary format), with possibility to export stl files (open formats) with Trios on Dental Desktop®
i-500®	Medit, Seoul, South Korea	3D in Motion Video Technology	obj, ply and stl (open formats)
EMERALD S®	Planmeca, Helsinki, Finland	Red, green and blue lasers- Projected Pattern Triangulation	3oxz (proprietary format), ply and stl (open formats)
EMERALD®		Red, green and blue lasers- Projected Pattern Triangulation	3oxz (proprietary format), ply and stl (open formats)
VIRTUOVIVO®	Dentalwings, Montreal, Canada	Blue laser-Multiscan Imaging Technology	xorder (proprietary format); ply, stl (open format)
DWIO®		Blue laser-Multiscan Imaging Technology	xorder (proprietary format); ply, stl (open format)
RUNEYES®	Runeyes MI, Ningbo, Zhejiang, China	Synchronous 3D Video Quick Technology	obj, ply and stl (open formats)

### Outcome variables

Three different evaluations were performed using the RM acquired with the desktop scanner and the models derived from the different IOSs: a mesh/mesh evaluation and a nurbs/nurbs evaluation, to compute the overall general trueness of the intraoral scanning models, and the evaluation of the linear and cross distances between the different SBs, for analysis of the local trueness of the intraoral scanning models. The latter evaluation used the STL files generated during the nurbs/nurbs evaluation. The evaluation of the overall trueness with the mesh/mesh and nurbs/nurbs methods (overall general trueness) was performed using reverse engineering software (Studio®, Geomagics, Morrisville, NC, USA) by the same experienced operator (FGM) who captured all the scans. The evaluation of the distances between the SBs was performed by another operator (MB) with many years of experience with 3D calculation software, using different software (Magics®, Materialise, Leuven, Belgium).

#### Mesh/mesh evaluation

This evaluation was based exclusively on the meshes (STL files) generated by scanning with the desktop scanner (RM) and the different IOSs, and took place as described in previous studies [14, 23, 24]. Each of the 10 meshes generated by each of the 12 IOSs was imported into reverse engineering software (Studio®, Geomagics, Morrisville, NC, USA), cut and trimmed with a single pre-formed template that included the pink gingiva area to be uniform in size, and then superimposed onto the RM captured with the desktop scanner, to evaluate the mean distance (± standard deviation, SD) between the models. The superimposition consisted of two steps. First, the software performed a rough alignment of the IOS model over the RM, determined by three or more points that were identified on the surface of the same SBs present in the two models. This first rough alignment was subsequently perfected by the software through the application of a best-fit algorithm that allowed the surfaces to fully overlap. The parameters for this last superimposition were set with a minimum of 100 iterations per case, and the surface registration was completed by a robust-iterative-closest-point (RICP) algorithm. With this RICP algorithm, the distances between the RM and the IOS models were minimised using a point-to-plane method and the congruence between corresponding structures was calculated. Finally, the signed mean ± SD of the distances in μm between the two superimposed models was calculated by the software, and a colorimetric map was generated to immediately visualise the distances between the models. The “3D deviation” function was used to generate the colorimetric map and quantify the distances between specific points, overall and in all space planes. The same setting for this map was used for all models, with the colour scale ranging from a maximum deviation of + 100 μm to − 100 μm, and the best result given by the deviations between + 30 μm and − 30 μm. The generated colour map indicated an outward (red) or inward (blue) deviation between the overlaid structures, while a minimal displacement (< 30 μm) was indicated by green. The data retrieved from these superimpositions for each IOS were saved in specific electronic datasheets (Excel®, Microsoft, Redmond, WA, USA) ready for statistical analysis, whereas the visual screenshots derived from each single superimposition were saved in another format (PowerPoint®, Microsoft, Redmond, WA, USA).

#### Nurbs/nurbs evaluation

This evaluation took place after replacing, within each mesh (the RM from the desktop scanner and all virtual models from the IOSs), the 6 SB meshes with the corresponding SB library file, downloaded from the official library of the implant manufacturer (Megagen®, Daegu, South Korea). A new STL file was saved for each virtual model, which included only 6 SBs (nurbs files) free in the space, representing the implant positions. These nurbs files were used for superimpositions.

In detail, each of the meshes, already cut and trimmed as previously described, was opened with reverse engineering software (Studio®, Geomagics, Morrisville, NC, USA). Then, 6 identical STL files were uploaded, those of the reference (nurbs) SB library file, provided directly from the implant library of the manufacturing company. At this point, these library files were superimposed onto each of the SBs present in the mesh, through the same superimposition procedure described above. First, a rough alignment of the library file was performed over the SB mesh; then, the surface superimposition was performed by the software, using the aforementioned RICP algorithm. This procedure was repeated for each single SB in the mesh, to obtain an STL file with the 6 SB library files in the correct position in the space. Then, the mesh was cancelled and a new file (nurbs file) with only the 6 SB library files in the proper position was saved. At the end of this procedure, which simulated what happens in the early stages of work in prosthetic CAD software (where the dental technician replaces the SB library file on the 3D reconstruction of the SB in the mesh, thus obtaining a hybrid model), it was possible to save files consisting of 6 SBs from the implant library free in the space, in a position derived from that of SBs in the mesh. These nurbs files were saved in special folders, labelled with the different IOS names and progressively numbered from 1 to 10, and were ready for analysis.

The analysis consisted of the superimposition of each of these nurbs STL files, derived from the different IOSs, over the RM nurbs. The procedure was the same as used in the mesh/mesh evaluation. Within the reverse engineering software (Studio®, Geomagics, Morrisville, NC, USA), the operator proceeded with an initial alignment of the nurbs file from the IOS onto the RM nurbs from the desktop scanner. This initial rough alignment took place by points, which were identified on the body of the scan abutments. Subsequently, the operator launched the best-fit algorithm, which perfected a surface alignment, generating the signed mean (± SD) of the distance in μm between the two nurbs files. Also in this case, the distances were represented graphically with a colorimetric map. The same settings used in the mesh/mesh evaluation were used, except for the green area, which was defined for an error < 25 μm. Once again, the data retrieved from these superimpositions for each IOS were saved in specific electronic datasheets (Excel®, Microsoft, Redmond, WA, USA) ready for statistical analysis, whereas the visual screenshots derived from each single superimposition were saved in another format (PowerPoint®, Microsoft, Redmond, WA, USA).

#### Linear and cross distances

**Fig. 2:**
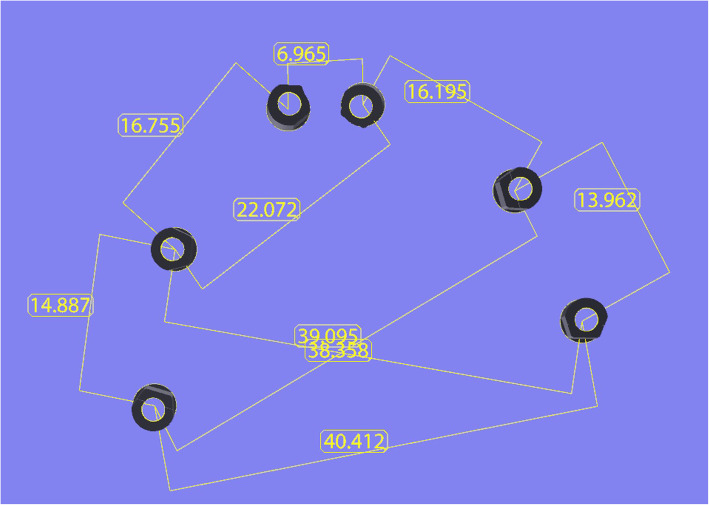
Automatic evaluation of the linear and cross distances with the reference file, in mm (Magics Magics®, Materialise, Leuven, Belgium)

A 3D calculation software (Magics®, Materialise, Leuven, Belgium) was used to compute the distances in μm between the different SBs. The calculation of the linear and cross distances was first performed on the RM nurbs file, to define the reference values for each distance. The following linear distances (i.e. distances of the SBs during the progression of the scan) were computed: S1–S2, S2–S3, S3–S4, S4–S5 and S5–S6. The cross distances (i.e. distances between SBs with different positions in the arch) were computed as follows: S1–S6, S1–S5, S2–S6 and S3–S5. The distances were automatically computed by the software as distances between the centroids at the bases of the SBs (Fig. 2). Once the reference values for each single distance were computed and saved in a specific datasheet (Excel®, Microsoft, Redmond, WA, USA), the same computation was repeated for each nurbs file from each single IOS scan. Tables were generated with all values for each scan taken from each IOS, and these values were used for statistical analysis.

### Statistical analysis

Data analysis and visualisation were performed using R (version 3.6.3) environment for statistical computing (R Foundation for Statistical Computing, Vienna, Austria). Descriptive statistics for quantitative variables were presented as medians (1st and 3rd quartiles; Tables 2 and 3) and medians (median absolute deviations; Table 4). Before regression modelling, mesh/mesh and nurbs/nurbs distance were log_10_-transformed (due to substantial right skewness on the raw scale); means and corresponding 95% confidence intervals (CI) were estimated using models then re-transformed to the raw scale (Tables 2 and 3). Linear and cross distances were used in raw scale. Linear models were used to estimate and compare mean mesh/mesh and nurbs/nurbs errors (on the log_10_-scale) between scanners. The Sandwich standard error estimator was used to address heteroskedasticity, and the Satterthwaite method was used to approximate degrees of freedom. The Tukey method (implemented in emmeans 1.4.5) was used to adjust *p*-values. A linear mixed effects model (implemented in lme4 1.1–21) was used to estimate and compare mean linear and cross distances (on raw scale) between scanners (to account hierarchy: scanner → pairs of SBs). The Sandwich cluster-robust variance-covariance matrix estimator was used to address heteroskedasticity, and the Satterthwaite method was again used to approximate degrees of freedom. The Tukey method was used to adjust p-values.

**Table 2:** Descriptive statistics (error in μm, medians and quartiles, means and 95% CIs) for mesh/mesh and nurbs/nurbs evaluations

Scanner	Mesh/Mesh		Nurbs/Nurbs	
	Median (Q_**1**_–Q_**3**_)	Mean (95% CI)	Median (Q_**1**_–Q_**3**_)	Mean (95% CI)
**CS 3600®**	35.5 (31.5–46.0)	36.5 [29.8; 44.6]	23.5 (21.5–34.0)	24.4 [18.0; 33.1]
**CS 3700®**	29.5 (27.2–34.5)	30.4 [26.7; 34.5]	22.0 (19.8–24.8)	21.9 [19.3; 25.0]
**DWIO®**	90.5 (84.2–110.8)	98.4 [84.4; 114.8]	65.0 (51.0–82.2)	69.9 [55.0; 88.9]
**EMERALD®**	76.0 (67.5–81.0)	76.1 [68.1; 85.1]	54.5 (40.8–60.5)	51.9 [43.5; 61.8]
**EMERALD S®**	51.0 (46.5–54.8)	52.9 [46.8; 59.7]	37.0 (31.2–40.8)	36.8 [31.1; 43.6]
**ITERO ELEMENTS 5D®**	32.0 (30.2–33.8)	31.4 [29.2; 33.8]	15.0 (14.2–16.8)	16.1 [12.9; 20.1]
**MEDIT I-500®**	31.5 (29.0–33.8)	32.2 [28.4; 36.6]	20.5 (17.5–25.8)	20.8 [16.9; 25.5]
**OMNICAM®**	80.5 (72.2–90.8)	79.6 [66.9; 94.6]	56.0 (33.2–62.5)	47.0 [33.7; 65.7]
**PRIMESCAN®**	39.5 (35.5–41.8)	38.4 [35.8; 41.2]	19.0 (17.0–23.8)	19.3 [16.3; 22.9]
**RUNEYES®**	41.5 (33.5–56.0)	44.4 [34.9; 56.5]	32.5 (26.0–43.0)	33.9 [26.4; 43.6]
**TRIOS 3®**	36.0 (35.2–38.5)	36.4 [33.9; 39.1]	20.5 (19.0–23.0)	20.2 [18.1; 22.7]
**VIRTUO VIVO®**	38.0 (35.2–42.2)	43.8 [33.6; 57.1]	28.0 (26.2–33.2)	32.0 [24.4; 42.0]

**Table 3:** Mesh/mesh evaluation. Differences (with standard errors) are presented at the top and right of the table, and correspond to row scanner names minus column scanner names. p-values for comparison are placed at the bottom and left of the table

	CS 3600®	CS 3700®	DWIO®	EMERALD®	EMERALD S®	ITERO ELEMENTS 5D®	MEDIT I-500®	OMNICAM®	PRIMESCAN®	RUNEYES®	TRIOS 3®	VIRTUO VIVO®
**CS 3600®**		0.08 (0.05)	−0.43 (0.06)	− 0.32 (0.05)	− 0.16 (0.05)	0.06 (0.05)	0.05 (0.05)	− 0.34 (0.06)	− 0.02 (0.05)	− 0.09 (0.07)	0.00 (0.05)	− 0.08 (0.07)
**CS 3700®**	0.9		−0.51 (0.04)	−0.40 (0.04)	− 0.24 (0.04)	−0.01 (0.03)	− 0.03 (0.04)	−0.42 (0.05)	− 0.10 (0.03)	−0.16 (0.06)	− 0.08 (0.03)	− 0.16 (0.06)
**DWIO®**	< 0.0001	< 0.0001		0.11 (0.04)	0.27 (0.04)	0.50 (0.04)	0.49 (0.04)	0.09 (0.05)	0.41 (0.04)	0.35 (0.06)	0.43 (0.04)	0.35 (0.07)
**EMERALD®**	< 0.0001	< 0.0001	0.2		0.16 (0.04)	0.38 (0.03)	0.37 (0.04)	−0.02 (0.05)	0.30 (0.03)	0.23 (0.06)	0.32 (0.03)	0.24 (0.06)
**EMERALD S®**	0.09	< 0.0001	< 0.0001	0.002		0.23 (0.03)	0.22 (0.04)	−0.18 (0.05)	0.14 (0.03)	0.08 (0.06)	0.16 (0.03)	0.08 (0.06)
**ITERO ELEMENTS 5D®**	1.0	1.0	< 0.0001	< 0.0001	< 0.0001		−0.01 (0.03)	−0.40 (0.04)	− 0.09 (0.02)	−0.15 (0.06)	− 0.06 (0.02)	−0.14 (0.06)
**MEDIT I-500®**	1.0	1.0	< 0.0001	< 0.0001	< 0.0001	1.0		−0.39 (0.05)	−0.08 (0.03)	− 0.14 (0.06)	−0.05 (0.03)	− 0.13 (0.06)
**OMNICAM®**	< 0.0001	< 0.0001	0.8	1.0	0.01	< 0.0001	< 0.0001		0.32 (0.04)	0.25 (0.07)	0.34 (0.04)	0.26 (0.07)
**PRIMESCAN®**	1.0	0.08	< 0.0001	< 0.0001	0.0009	0.007	0.4	< 0.0001		−0.06 (0.06)	0.02 (0.02)	−0.06 (0.06)
**RUNEYES®**	1.0	0.2	< 0.0001	0.006	1.0	0.2	0.5	0.009	1.0		0.09 (0.06)	0.01 (0.08)
**TRIOS 3®**	1.0	0.4	< 0.0001	< 0.0001	< 0.0001	0.2	0.9	< 0.0001	1.0	0.9		−0.08 (0.06)
**VIRTUO VIVO®**	1.0	0.4	< 0.0001	0.01	1.0	0.4	0.6	0.02	1.0	1.0	1.0

**Table 4:** Nurbs/nurbs evaluation. Differences (with standard errors) are presented at the top and right of the table, and correspond to row scanner names minus column scanner names. *p*-values for comparison are placed at the bottom and left of the table

	CS 3600®	CS 3700®	DWIO®	EMERALD®	EMERALD S®	ITERO ELEMENTS 5D®	MEDIT I-500®	OMNICAM®	PRIMESCAN®	RUNEYES®	TRIOS 3®	VIRTUO VIVO®
**CS 3600®**		0.05 (0.07)	−0.46 (0.09)	−0.33 (0.08)	− 0.18 (0.08)	0.18 (0.08)	0.07 (0.08)	−0.28 (0.10)	0.10 (0.08)	−0.14 (0.09)	0.08 (0.07)	−0.12 (0.09)
**CS 3700®**	1.0		−0.50 (0.06)	−0.37 (0.05)	− 0.23 (0.05)	0.13 (0.06)	0.02 (0.05)	−0.33 (0.08)	0.05 (0.05)	−0.19 (0.06)	0.03 (0.04)	−0.16 (0.07)
**DWIO®**	< 0.0001	< 0.0001		0.13 (0.07)	0.28 (0.06)	0.64 (0.07)	0.53 (0.07)	0.17 (0.09)	0.56 (0.06)	0.31 (0.08)	0.54 (0.06)	0.34 (0.08)
**EMERALD®**	0.003	< 0.0001	0.7		0.15 (0.05)	0.51 (0.06)	0.40 (0.06)	0.04 (0.08)	0.43 (0.05)	0.18 (0.07)	0.41 (0.05)	0.21 (0.07)
**EMERALD S®**	0.5	0.0003	0.002	0.2		0.36 (0.06)	0.25 (0.06)	−0.11 (0.08)	0.28 (0.05)	0.04 (0.07)	0.26 (0.04)	0.06 (0.07)
**ITERO ELEMENTS 5D®**	0.6	0.4	< 0.0001	< 0.0001	< 0.0001		−0.11 (0.07)	−0.47 (0.09)	− 0.08 (0.06)	−0.32 (0.07)	− 0.10 (0.05)	−0.30 (0.08)
**MEDIT I-500®**	1.0	1.0	< 0.0001	< 0.0001	0.002	0.9		−0.35 (0.09)	0.03 (0.06)	−0.21 (0.07)	0.01 (0.05)	−0.19 (0.07)
**OMNICAM®**	0.2	0.003	0.8	1.0	1.0	< 0.0001	0.004		0.39 (0.08)	0.14 (0.09)	0.37 (0.08)	0.17 (0.09)
**PRIMESCAN®**	1.0	1.0	< 0.0001	< 0.0001	< 0.0001	1.0	1.0	0.0005		−0.24 (0.07)	−0.02 (0.05)	−0.22 (0.07)
**RUNEYES®**	0.9	0.1	0.004	0.2	1.0	0.001	0.1	0.9	0.02		0.22 (0.06)	0.03 (0.08)
**TRIOS 3®**	1.0	1.0	< 0.0001	< 0.0001	< 0.0001	0.8	1.0	0.0004	1.0	0.01		−0.20 (0.06)
**VIRTUO VIVO®**	1.0	0.4	0.002	0.1	1.0	0.009	0.3	0.8	0.09	1.0	0.10	

## Results

**Fig. 3:**
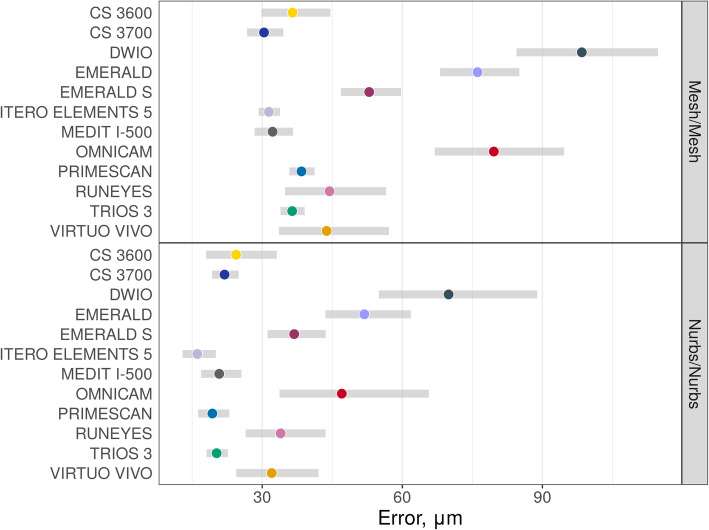
Estimated mean errors (in μm, with 95% CIs) for mesh/mesh and nurbs/nurbs evaluations

**Fig. 4:**
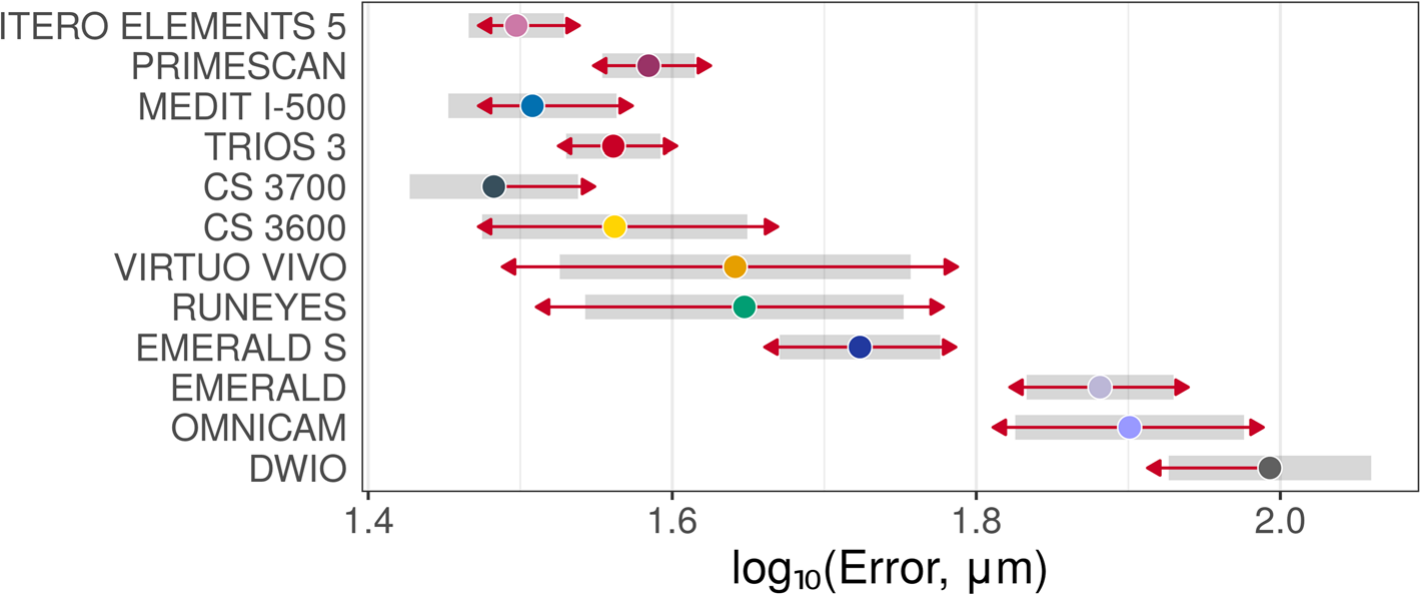
Overall mesh/mesh evaluation. A log10 scale was used for the analysis. Log transformed values were represented on the x-axis. The overlap of red arrows between pairs of scanners indicates no statistically significant difference

**Fig. 5:**
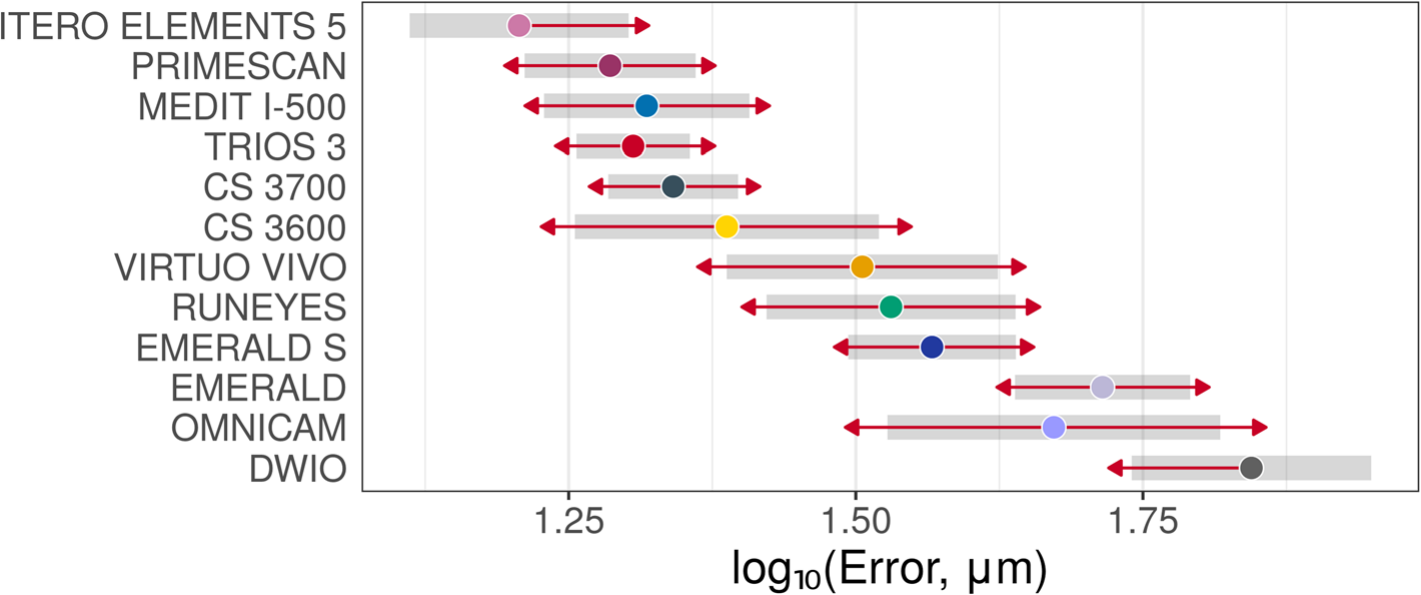
Overall nurbs/nurbs evaluation. A log10 scale was used for the analysis. Log transformed values were represented on the x-axis. The overlap of red arrows between pairs of scanners indicates no statistically significant difference

Descriptive statistics (medians and quartiles; means and 95% CIs) for mesh/mesh and nurbs/nurbs errors are presented in Table 2. Estimated mean errors (with 95% CIs – symmetrical on the log scale) on raw scales are presented in Figs. 3, 4 and 5. In thes latter two figures, the overlap of the red arrows between pairs of scanners indicates no statistically significant difference. A statistically significant difference was found among different scanner pairs.

**Fig. 6:**
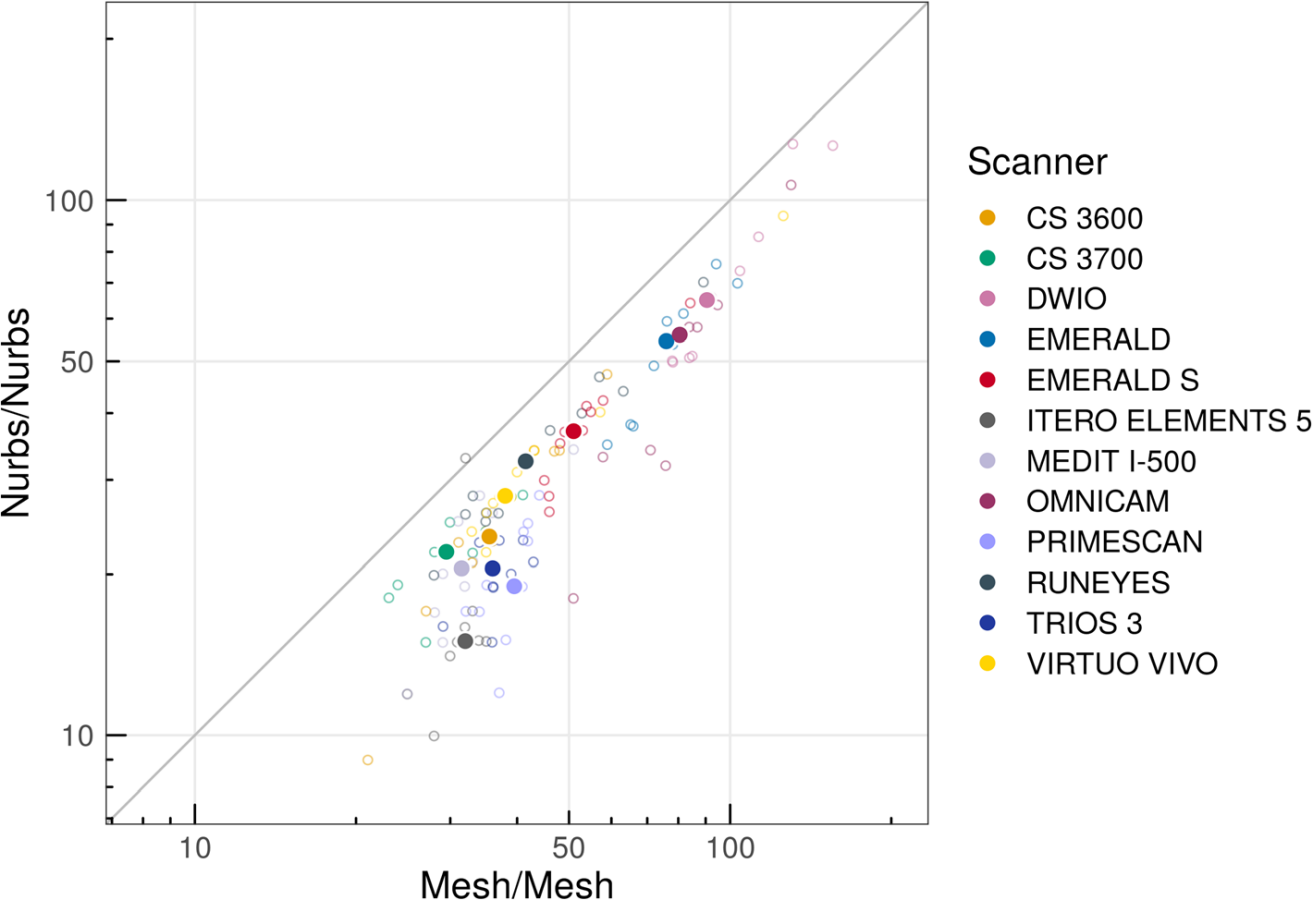
Circles correspond to individual observations, filled dots – medians for each scanner. The scatter plot highlighted that nurbs/nurbs errors were systematically lower than mesh/mesh errors

Nurbs/nurbs errors were systematically lower than mesh/mesh errors, as evidenced in the scatter plot in Fig. 6, as the test result for H0: β_1_ = 1 (corresponds to model: nurbs/nurbs = β_0_ [different for each scanner – random intercept] + 1 × mesh/mesh): t = 10.7, non-centrality parameter = 1, df = 87.6, *p* < 0.0001. β_1_ = 1.21 (95% CI 1.10; 1.33). Pairwise differences in errors (on log_10_-scale/orders of differences with corresponding standard errors and *p*-values) with the different methods are presented in Tables 3 and 4.

**Fig. 7:**
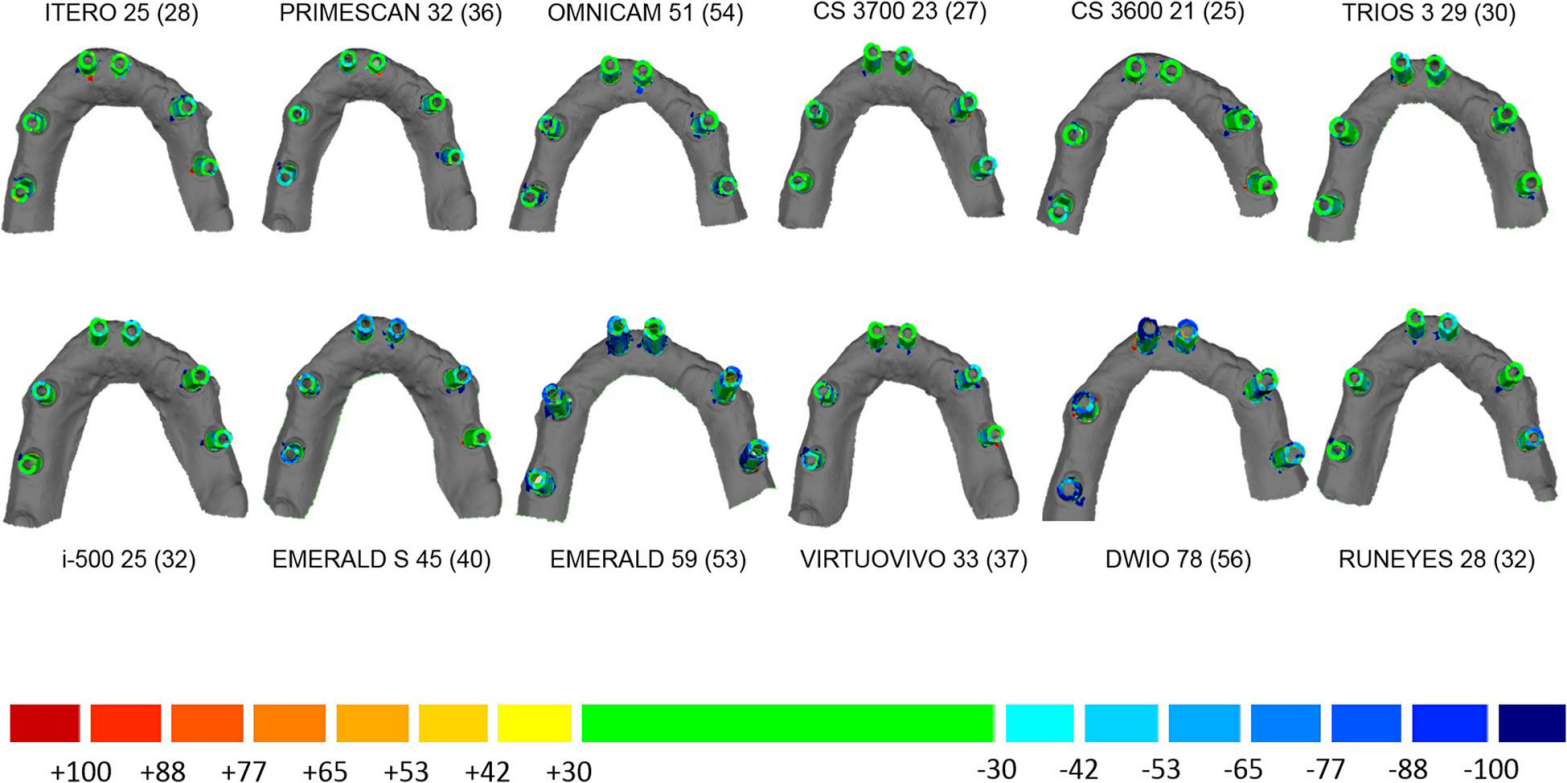
The best single results (mean ± SD) obtained by each IOS with the mesh/mesh method, in μm

With the mesh/mesh method, the best results were obtained by CS 3700® (mean error 30.4 μm; 95% CI 26.7–34.5 μm) followed by ITERO ELEMENTS 5D® (mean error 31.4 μm; 95% CI 29.2–33.8 μm), i-500® (mean error 32.2 μm; 95% CI 28.4–36.6 μm); TRIOS 3® (mean error 36.4 μm; 95% CI 33.9–39.1 μm), CS 3600® (mean error 36.5 μm; 95% CI 29.8–44.6 μm), PRIMESCAN® (mean error 38.4 μm; 95% CI 35.8–41.2 μm), VIRTUO VIVO® (mean error 43.8 μm; 95% CI 33.6–57.1 μm), RUNEYES® (mean error 44.4 μm; 95% CI 34.9–56.5 μm), EMERALD S® (mean error 52.9 μm; 95% CI 46.8–59.7 μm), EMERALD® (mean error 76.1 μm; 95% CI 68.1–85.1 μm), OMNICAM® (mean error 79.6 μm; 95% CI 66.9–94.6 μm) and DWIO® (mean error 98.4 μm; 95% CI 84.4–114.8 μm). The best single results obtained by each IOS with the mesh/mesh method were summarized in Fig. 7. Statistically significant differences were found between the IOSs, as reported at the bottom and left of Table 3.

**Fig. 8:**
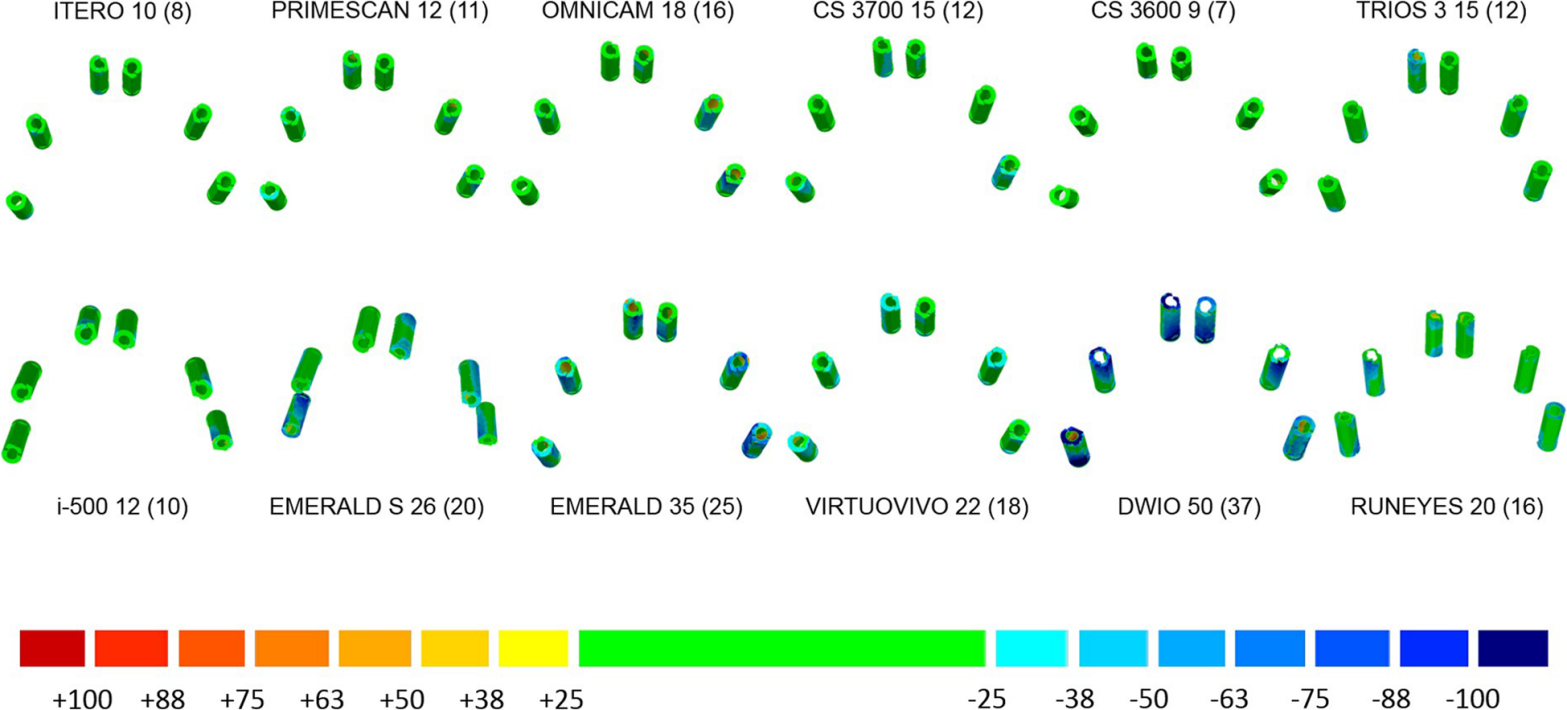
The best single results (mean ± SD) obtained by each IOS with the nurbs/nurbs method, in μm

With the nurbs/nurbs method, the best results were obtained by ITERO ELEMENTS 5D® (mean error 16.1 μm; 95% CI 12.9–20.1 μm), followed by PRIMESCAN® (19.3 μm; 95% CI 16.3–22.9 μm), TRIOS 3® (mean error 20.2 μm; 95% CI 18.1–22.7 μm), i-500® (mean error 20.8 μm; 95% CI 16.9–25.5 μm), CS 3700® (mean error 21.9 μm; 95% CI 19.3–25.0 μm), CS 3600® (mean error 24.4 μm; 95% CI 18.0–33.1 μm), VIRTUO VIVO® (mean error 32.0 μm; 95% CI 24.4–42.0 μm), RUNEYES® (mean error 33.9 μm; 95% CI 26.4–43.6 μm), EMERALD S® (mean error 36.8 μm; 95% CI 31.1–43.6 μm), OMNICAM® (mean error 47.0 μm; 95% CI 33.7–65.7 μm), EMERALD® (mean error 51.9 μm; 95% CI 43.5–61.8 μm) and DWIO® (mean error 69.9 μm; 95% CI 55.0–88.9 μm). The best single results obtained by each IOS with the nurbs/nurbs method were summarized in Fig. 8. Statistically significant differences were found between the IOSs, as reported at the bottom and left of Table 4.

**Fig. 9:**
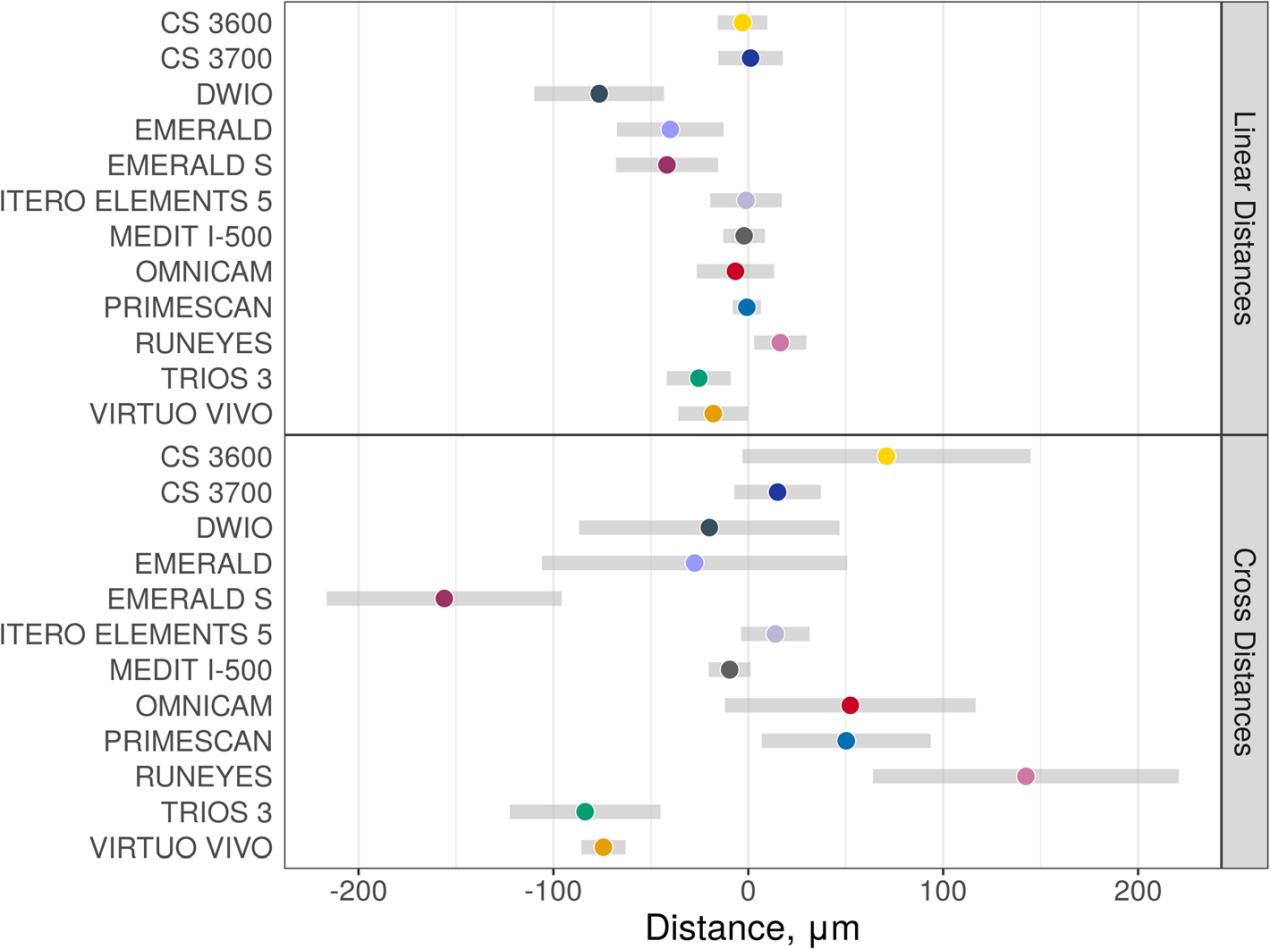
Estimated mean errors (in μm, with 95% CIs) for linear and cross distances

**Fig. 10:**
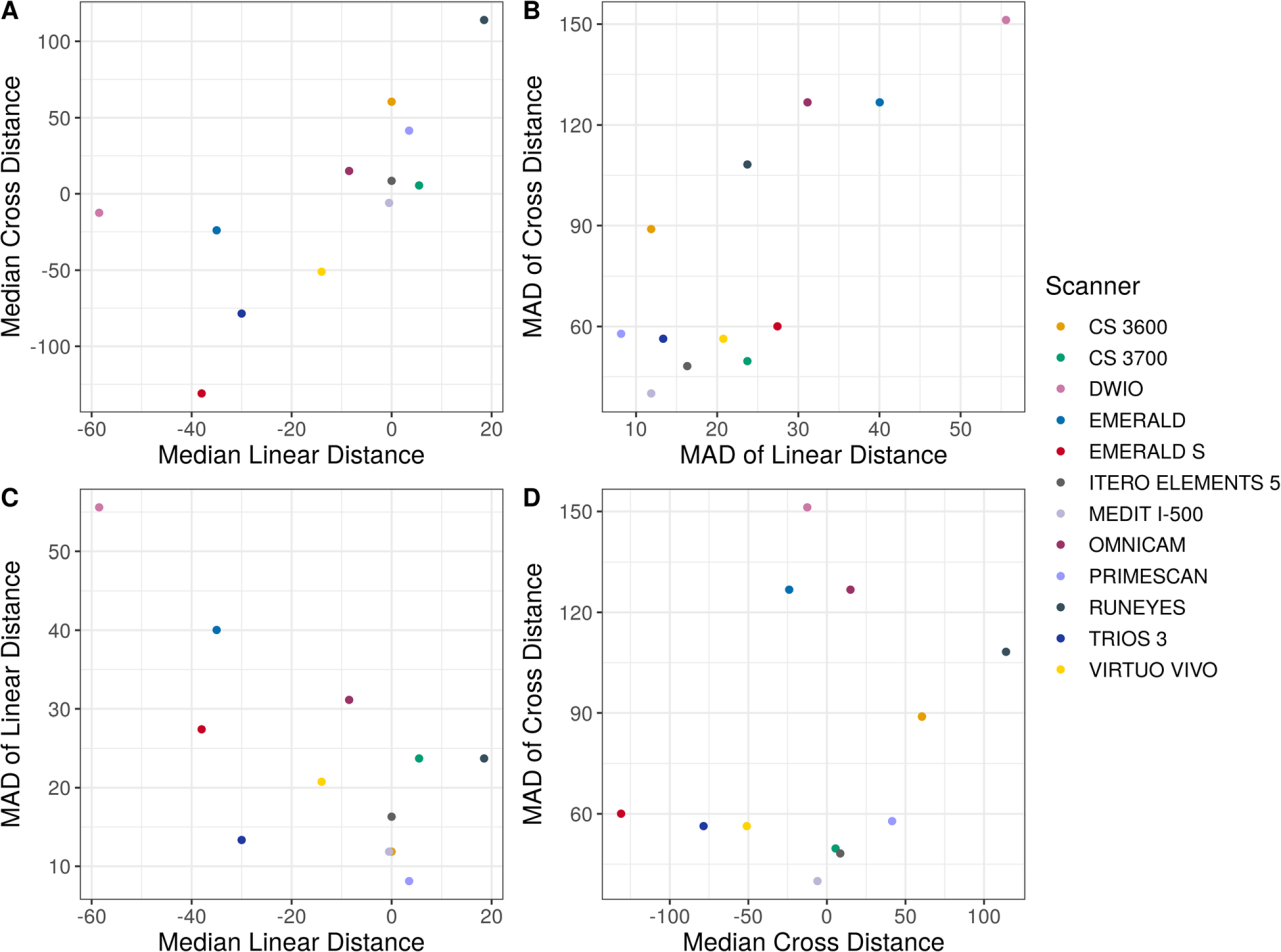
Medians and median absolute deviations (from median, MAD)

**Fig. 11:**
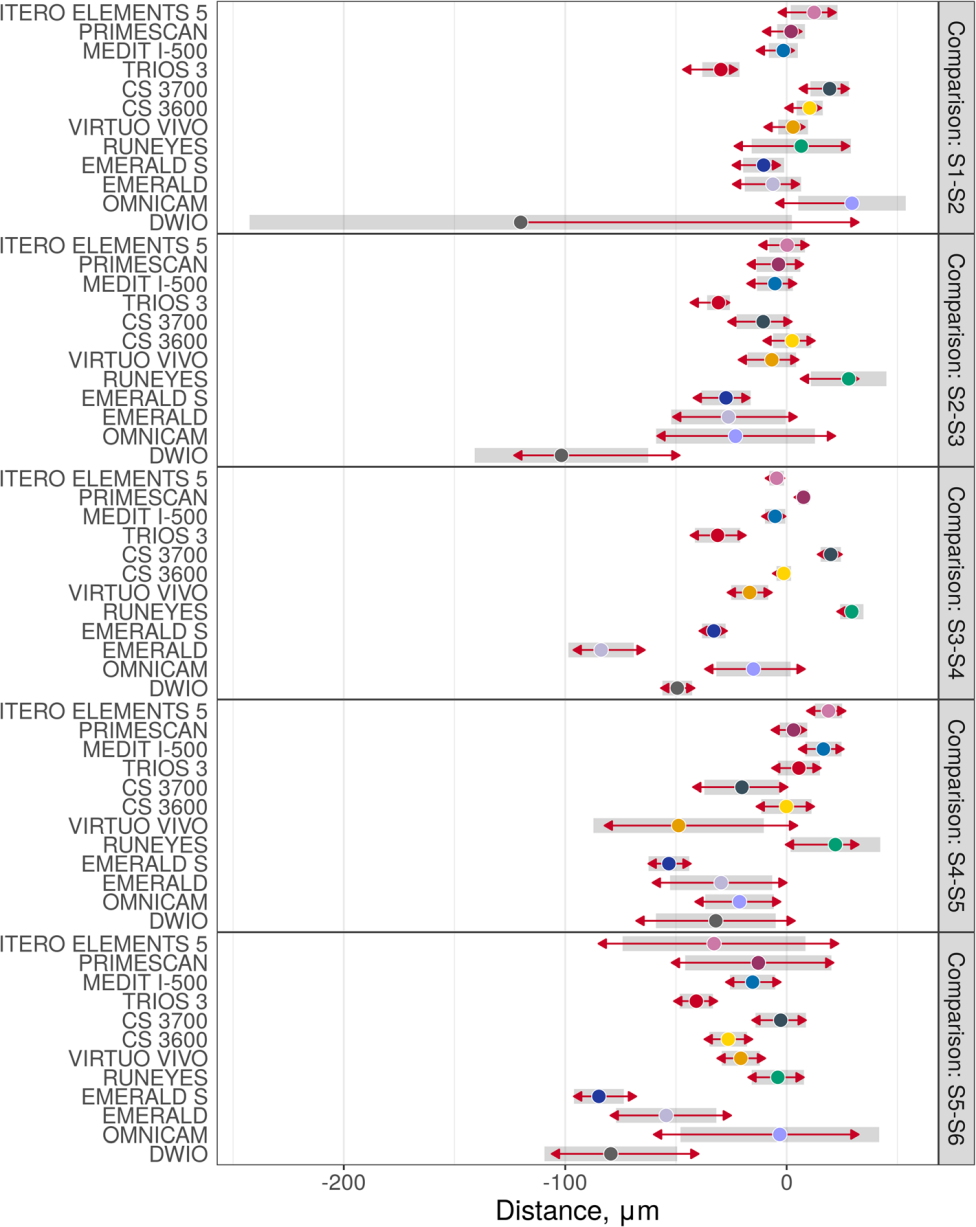
Linear distances: mean error (CI 95%) and comparison between the different intraoral scanners. The overlap of the red arrows between pairs of scanners indicates no statistically significant difference

**Fig. 12:**
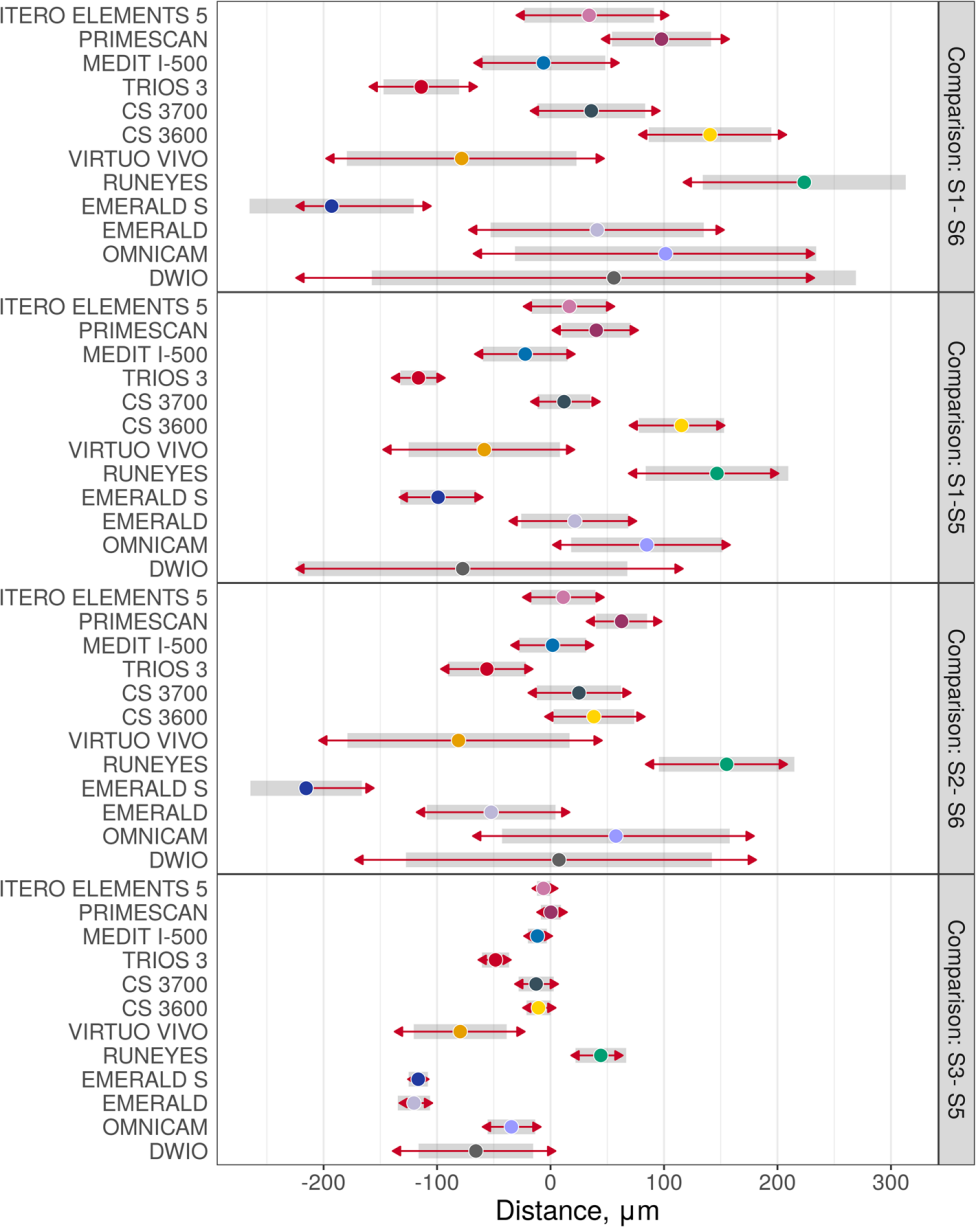
Cross distances: mean error (CI 95%) and comparison between the different intraoral scanners. The overlap of the red arrows between pairs of scanners indicates no statistically significant difference

Descriptive statistics (medians and quartiles, means and 95% CIs) for linear and cross distances are presented in Table 5. Estimated mean distances (with 95% CIs) on raw scales are presented in Fig. 9. Pairwise differences in distances (on raw scales with corresponding standard errors and *p*-values) are presented in Tables 6 and 7; significant differences were found between the IOSs, as reported at the bottom and left of the tables. Medians and median absolute deviations (from median, MAD) are presented in Table 8 and Fig. 10. Correlations were found between these values: medians of linear and cross distances (0.80, 95% CI 0.41–0.94, *p* = 0.0019), MADs of linear and cross distances (0.66, 95% CI 0.14–0.90, *p* = 0.019), medians and MADs of linear distances (− 0.52, 95% CI -0.84–0.07, *p* = 0.082), and medians and MADs of cross distances (0.13, 95% CI -0.48–0.65, *p* = 0.696). Finally, linear and cross distances were evaluated for each of the SB pairs, as reported in Figs. 11 and 12, respectively. In these figures, the overlap of the red arrows between pairs of scanners indicates no statistically significant difference. Once again, a statistically significant difference was found among different scanner pairs.

**Table 5:** Descriptive statistics (error in μm, medians and quartiles, means and 95% CIs) for linear and cross distances

Scanner	Linear distances			Cross distances		
	Median (Q_**1**_–Q_**3**_)^**a**^	Median (Q_**1**_–Q_**3**_)^**b**^	Mean (95% CI)^**c**^	Median (Q_**1**_–Q_**3**_)^**a**^	Median (Q_**1**_–Q_**3**_)^**b**^	Mean (95% CI)^**c**^
CS 3600®	0.0 (−9.2–8.0)	8.0 (4.0–19.0)	−3.0 [−15.7; 9.7]	60.5 (−2.5–109.2)	62.5 (19.2–109.2)	70.9 [−3.0; 144.8]
CS 3700®	5.5 (−15.8–19.5)	19.0 (10.0–26.8)	1.2 [− 15.3; 17.7]	5.5 (−25.0–39.2)	35.0 (20.5–50.0)	15.0 [−7.2; 37.2]
DWIO®	− 58.5 (− 104.5–-36.2)	58.5 (36.2–104.5)	−76.5 [− 109.8; −43.3]	−12.5 (− 119.0–55.5)	111.0 (46.5–230.2)	− 20.0 [−86.8; 46.8]
EMERALD®	−35.0 (−69.8–-11.8)	41.0 (16.0–69.8)	−40.1 [−67.4; − 12.7]	− 24.0 (− 112.8–40.8)	103.0 (34.5–122.0)	−27.6 [− 105.9; 50.8]
EMERALD S®	− 38.0 (− 51.8–-19.2)	38.0 (19.2–51.8)	−41.7 [− 67.9; − 15.6]	− 131.0 (− 220.8–-97.2)	131.0 (97.2–220.8)	− 156.0 [− 216.3; − 95.7]
ITERO ELEMENTS 5D®	0.0 (−6.0–13.0)	11.0 (4.0–17.8)	−1.2 [− 19.5; 17.2]	8.5 (− 18.2–46.8)	36.0 (14.5–57.2)	13.9 [− 3.7; 31.5]
MEDIT I-500®	− 0.5 (− 11.5–5.8)	8.0 (3.0–16.8)	−2.2 [− 12.9; 8.6]	− 6.0 (− 27.5–23.5)	27.0 (15.5–54.0)	−9.6 [− 20.3; 1.2]
OMNICAM®	−8.5 (− 30.5–10.5)	23.0 (9.8–52.0)	−6.6 [− 26.5; 13.3]	15.0 (−48.5–138.2)	88.5 (27.5–150.2)	52.3 [− 12.0; 116.7]
PRIMESCAN®	3.5 (− 2.0–9.0)	6.5 (3.0–11.0)	−0.8 [− 8.0; 6.4]	41.5 (7.0–86.8)	41.5 (14.8–86.8)	50.2 [6.9; 93.6]
RUNEYES®	18.5 (− 1.0–33.2)	23.0 (15.0–34.8)	16.4 [3.0; 29.8]	114.0 (49.2–216.8)	114.0 (49.2–216.8)	142.4 [64.0; 220.9]
TRIOS 3®	−30.0 (− 37.0–-19.2)	30.0 (22.0–37.0)	−25.4 [− 41.8; −9.0]	− 78.5 (− 124.2–-50.0)	78.5 (56.0–124.2)	− 83.7 [− 122.4; − 45.1]
VIRTUO VIVO®	−14.0 (− 25.5–0.5)	15.5 (6.2–25.5)	− 18.0 [− 35.9; − 0.1]	−51.0 (− 85.5–-11.0)	55.5 (30.2–87.8)	−74.4 [− 85.7; − 63.0]

^a^Median (interquartile range) error calculated on raw data

^b^Median (interquartile range) absolute error

^c^Mean error (95% CI) estimated using linear mixed effects models

**Table 6:** Linear distances. Differences (with standard errors) are presented at the top and right of the table, and correspond to row scanner names minus column scanner names. *p*-values for comparison are placed at the bottom and left of the table

	CS 3600®	CS 3700®	DWIO®	EMERALD®	EMERALD S®	ITERO ELEMENTS 5D®	MEDIT I-500®	OMNICAM®	PRIMESCAN®	RUNEYES®	TRIOS 3®	VIRTUO VIVO®
**CS 3600®**		−4.1 (8.9)	73.5 (18.7)	37.1 (11.6)	38.8 (7.2)	−1.8 (4.5)	− 0.8 (5.5)	3.6 (10.3)	−2.2 (4.2)	−19.4 (6.0)	22.4 (8.2)	15.0 (9.1)
**CS 3700®**	1.0		77.7 (20.5)	41.2 (17.2)	42.9 (11.4)	2.3 (12.6)	3.3 (11.3)	7.8 (7.7)	1.9 (7.5)	−15.2 (10.9)	26.6 (14.2)	19.2 (6.8)
**DWIO®**	0.005	0.009		−36.5 (26.3)	−34.8 (24.8)	−75.4 (17.8)	−74.4 (14.4)	−69.9 (23.7)	− 75.8 (15.4)	−92.9 (15.0)	−51.1 (13.0)	−58.5 (24.3)
**EMERALD®**	0.06	0.4	1.0		1.7 (14.3)	−38.9 (11.4)	−37.9 (12.5)	− 33.5 (12.5)	−39.3 (14.2)	−56.5 (16.1)	−14.7 (13.6)	−22.1 (14.2)
**EMERALD S®**	< 0.0001	0.010	1.0	1.0		−40.6 (9.4)	−39.6 (12.6)	−35.1 (13.1)	−41.0 (10.8)	−58.1 (11.2)	−16.3 (14.9)	−23.7 (10.4)
**ITERO ELEMENTS 5D®**	1.0	1.0	0.002	0.03	0.001		1.0 (5.1)	5.4 (12.8)	− 0.4 (6.8)	−17.6 (7.9)	24.2 (6.1)	16.8 (13.4)
**MEDIT I-500®**	1.0	1.0	< 0.0001	0.1	0.08	1.0		4.4 (12.0)	−1.4 (4.2)	−18.6 (6.4)	23.2 (3.1)	15.8 (12.7)
**OMNICAM®**	1.0	1.0	0.1	0.2	0.2	1.0	1.0		−5.8 (10.4)	−23.0 (14.7)	18.8 (14.5)	11.4 (8.4)
**PRIMESCAN®**	1.0	1.0	< 0.0001	0.2	0.008	1.0	1.0	1.0		−17.2 (4.8)	24.6 (7.1)	17.2 (9.7)
**RUNEYES®**	0.06	1.0	< 0.0001	0.02	< 0.0001	0.5	0.1	0.9	0.02		41.8 (8.2)	34.4 (11.7)
**TRIOS 3®**	0.2	0.8	0.005	1.0	1.0	0.005	< 0.0001	1.0	0.03	< 0.0001		−7.4 (15.7)
**VIRTUO VIVO®**	0.9	0.2	0.4	0.9	0.5	1.0	1.0	1.0	0.8	0.1	1.0

**Table 7:** Cross distances. Differences (with standard errors) are presented at the top and right of the table, and correspond to row scanner names minus column scanner names. *p*-values for comparison are placed at the bottom and left of the table

	CS 3600®	CS 3700®	DWIO®	EMERALD®	EMERALD S®	ITERO ELEMENTS 5D®	MEDIT I-500®	OMNICAM®	PRIMESCAN®	RUNEYES®	TRIOS 3®	VIRTUO VIVO®
**CS 3600®**		55.9 (27.9)	90.9 (35.7)	98.4 (4.2)	226.9 (47.3)	57.0 (27.2)	80.5 (36.4)	18.6 (13.0)	20.6 (23.2)	−71.6 (18.5)	154.6 (52.6)	145.2 (32.6)
**CS 3700®**	0.7		35.0 (23.4)	42.6 (29.5)	171.0 (36.8)	1.1 (4.6)	24.6 (9.4)	−37.3 (21.6)	−35.2 (10.2)	− 127.4 (26.9)	98.8 (25.4)	89.4 (12.1)
**DWIO®**	0.3	0.9		7.5 (36.8)	136.0 (58.1)	−33.9 (26.3)	−10.4 (28.0)	−72.3 (30.2)	−70.3 (16.6)	− 162.5 (23.8)	63.7 (39.2)	54.3 (34.8)
**EMERALD®**	< 0.0001	1.0	1.0		128.5 (49.9)	−41.4 (29.3)	−18.0 (38.3)	−79.9 (11.5)	− 77.8 (24.4)	− 170.0 (17.3)	56.2 (54.4)	46.8 (34.6)
**EMERALD S®**	0.0001	0.0003	0.5	0.3		−169.9 (32.8)	− 146.4 (33.2)	− 208.3 (48.4)	− 206.3 (45.4)	− 298.5 (58.3)	−72.3 (36.1)	−81.7 (25.0)
**ITERO ELEMENTS 5D®**	0.6	1.0	1.0	1.0	< 0.0001		23.5 (9.3)	−38.4 (22.8)	−36.4 (13.0)	−128.5 (29.0)	97.6 (25.4)	88.3 (9.1)
**MEDIT I-500®**	0.5	0.3	1.0	1.0	0.0008	0.3		−61.9 (30.8)	−59.8 (18.8)	− 152.0 (36.0)	74.2 (16.3)	64.8 (10.1)
**OMNICAM®**	1.0	0.9	0.4	< 0.0001	0.001	0.9	0.7		2.1 (16.4)	−90.1 (12.9)	136.1 (46.5)	126.7 (28.7)
**PRIMESCAN®**	1.0	0.03	0.002	0.07	0.0004	0.2	0.07	1.0		−92.2 (17.4)	134.0 (34.2)	124.6 (21.8)
**RUNEYES®**	0.007	0.0002	< 0.0001	< 0.0001	< 0.0001	0.0007	0.002	< 0.0001	< 0.0001		226.2 (51.5)	216.8 (37.0)
**TRIOS 3®**	0.1	0.007	0.9	1.0	0.7	0.008	0.0004	0.1	0.006	0.0008		−9.4 (22.1)
**VIRTUO VIVO®**	0.0006	< 0.0001	0.9	1.0	0.05	< 0.0001	< 0.0001	0.0008	< 0.0001	< 0.0001	1.0

**Table 8:** Medians and median absolute deviations (from median, MAD)

Scanner	Linear distances median (MAD)	Cross distances median (MAD)
**CS 3600®**	0.0 (11.9)	60.5 (89.0)
**CS 3700®**	5.5 (23.7)	5.5 (49.7)
**DWIO®**	−58.5 (55.6)	−12.5 (151.2)
**EMERALD®**	−35.0 (40.0)	−24.0 (126.8)
**EMERALD S®**	−38.0 (27.4)	−131.0 (60.0)
**ITERO ELEMENTS 5®**	0.0 (16.3)	8.5 (48.2)
**MEDIT I-500®**	−0.5 (11.9)	−6.0 (40.0)
**OMNICAM®**	−8.5 (31.1)	15.0 (126.8)
**PRIMESCAN®**	3.5 (8.2)	41.5 (57.8)
**RUNEYES®**	18.5 (23.7)	114.0 (108.2)
**TRIOS 3®**	−30.0 (13.3)	−78.5 (56.3)
**VIRTUO VIVO®**	−14.0 (20.8)	−51.0 (56.3)

## Discussion

To date, few clinical studies have supported the use of optical impression for manufacturing FA restorations in the completely edentulous patient; these studies are limited to the restorations of patients with 4 implants [30–32]. In more complex clinical situations, with FA supported by between 6 and 8 implants, the scientific literature has not yet validated the use of optical impression [16, 17, 33]. For this reason, and since the technological development of IOSs is constant through the improvement and implementation of new software and hardware, constant updates are needed on the accuracy of the scanners on the market.

In recent years, the scientific literature has attempted to investigate the accuracy of IOSs to extend the use of the optical impression to complex clinical applications, such as capturing impressions for modelling and manufacturing FA restorations via a full digital workflow [14, 15, 19–26, 29].

Most studies investigating the trueness of optical impressions that are currently available in the scientific literature have used a mesh/mesh approach, superimposing IOS models onto an RM using best-fit algorithms, and these functions align the meshes to assess the minimum error [14, 15, 23, 24, 29]. As a consequence, the error is distributed homogeneously throughout the whole mesh [14, 15, 29]. This method is valid to assess the overall trueness because it is immediate and not affected by other variables (tolerances in the fabrication of the SBs, for example) [15, 29]. However, in implant-supported restorations, it is also important to assess the error between fixation points; even a single error localised in one point can determine a clinical misfit of the prosthetic structure. Some studies have therefore attempted to assess distance or angulation errors between fixation points [25, 26]. To make linear measurements that are reliable, however, it is necessary to work on library files (or nurbs files), on which specific landmarks (such as the centroids) can be precisely and automatically identified by the software. Working with nurbs files also allows more faithfully replicating what happens clinically in the early stages of prosthetic CAD [27].

Our present in vitro study therefore used three different methods to investigate the trueness of 12 IOSs in the FA implant impression: a mesh/mesh method and a nurbs/nurbs method to evaluate overall trueness, and the computation of linear and cross distances between the SBs to evaluate local trueness. In our study, statistically significant differences were found between the different IOSs, as previously reported [14, 15, 19–26, 29]. In particular, the mesh/mesh and nurbs/nurbs analysis have allowed identifying three groups of scanners, characterised by different levels of trueness. The first group, comprising the IOSs with the highest accuracy, consisted of ITERO ELEMENTS 5D®, PRIMESCAN®, CS 3700®, CS 3600®, TRIOS3® and i-500®. These scanners have an average intrinsic error < 40 μm with the mesh/mesh method and < 25 μm with the nurbs/nurbs method and represent a theoretically compatible solution for taking impressions for FA restorations. The second group of scanners presented positive results, although probably still not compatible with the capture of a FA impression. These were EMERALD S®, EMERALD®, OMNICAM®, VIRTUO VIVO® and RUNEYES®, which presented an average intrinsic error between 40 and 80 μm with the mesh/mesh method and between 25 and 50 μm with the nurbs/nurbs method. DWIO® remained distanced from all the others, with an intrinsic error > 80 μm in the mesh/mesh analysis and > 50 μm in the nurbs/nurbs analysis, certainly incompatible with the FA impression. The data of the overall trueness were confirmed by the analysis of the local distances between the SBs, i.e. the linear and cross distances, which again highlighted the existence of three groups of IOSs in this study, characterised by different performances. In fact, linear error analysis along the scan revealed higher reliability for ITERO ELEMENTS 5D®, PRIMESCAN®, CS 3700®, CS 3600®, TRIOS3® and i-500®, which showed lower errors than other IOSs. For the particular scanning strategy used in this study, it was not possible to evaluate in detail, for each scanner, the percentage growth of the error as the scan proceeded; however, it was evident that greater variability was present in the paths S2-S3 and S4-S5, corresponding to the area of greatest curvature of the physical model. This finding must be confirmed by further studies, but it would seem to indicate difficulty for IOSs in accurately detecting stretches of curvature. The evaluation of cross measurements naturally resulted in larger errors, in direct proportion to the actual distance between the SBs. Here, too, the evaluation showed significant differences between the different IOSs.

The main advantage of this study is in having compared 12 IOSs, and done so using different techniques, to understand the intrinsic trueness of the scanners at a global and local level by measuring the distances between the different SBs. On one hand, the evaluation with the mesh/mesh method has the advantage of directly highlighting the quality of the scan. In this study, with the mesh/mesh analysis, the best absolute performance was obtained by CS 3700® (mean error 30.4 μm; 95% CI 26.7–34.5 μm) followed by ITERO ELEMENTS 5D® (mean error 31.4 μm; 95% CI 29.2–33.8 μm) and i − 500® (mean error 32.2 μm; 95% CI 28.4–36.6 μm). These IOSs were the best in the representation of the SBs and the tissues around them, although in an in vitro study, the soft tissues are a resin copy of the real human gums. On the other hand, the nurbs/nurbs evaluation allows replicating what happens clinically, when within the CAD software the meshes of the SBs are replaced with the corresponding library file, generating a hybrid virtual model on which the modelling takes place. This approach specifically assesses the sole trueness of the position of the implants after the mesh/nurbs replacement in CAD, without any interference from the soft tissues; it is also prerequisite for the correct implementation of the analysis of the distances between the SBs, carried out as an evaluation of the distances between the centroids at their bases. In the analysis of the overall trueness with the nurbs/nurbs method, the best results were obtained by ITERO ELEMENTS 5D® (mean error 16.1 μm; 95% CI 12.9–20.1 μm), followed by PRIMESCAN® (19.3 μm; 95% CI 16.3–22.9 μm) and TRIOS 3® (mean error 20.2 μm; 95% CI 18.1–22.7 μm). These results reflect a very low error in the position of the SBs with these IOSs, which could certainly be considered compatible, in all cases, with the realisation of a FA restoration via a full digital workflow. The evaluation of the distances between the single SBs confirmed these positive results, particularly with regard to the linear distances (distances along the arch). Obviously, as expected, the local errors tended to grow in the cross distances, particularly between the most distal SBs, but this error is certainly contained compared to what was described only a few years ago in similar studies [25, 33].

However, the data presented in this study, which refer to the intrinsic trueness of the different IOSs analysed, must be taken with caution. The IOS is not the only factor involved in determining the final accuracy of an optical impression: the operator [34], patient [35], light conditions [36] and SB [37–40] are also key. The operator is essential because different scanning strategies and different levels of experience can determine different results, as reported in the literature [34]. In the present study, all models were captured by the same operator with many years of experience in intraoral scanning; however, the choice of scan strategy may have favoured some IOSs over others. To date, unfortunately, little is known about the effects of different scanning strategies, since the scientific literature on this topic is scarce [2, 34], and even the manufacturers have not clarified this aspect in full. The patient is equally important. Implants can be inserted in different positions, inclinations and depths, and these factors can positively (or negatively) influence the final trueness of the scan [35]. With regard to this aspect, the literature is scarce too [16, 33], and investigating more deeply the effects of these variables is advisable. In the present study, the SBs were rather parallel to each other, simulating an ideal condition with implants placed after a guided surgery procedure; this condition can be considered ideal but is not always found in clinical practice. Light conditions are another factor of great importance in intraoral scanning [36]. In the present in vitro study, all scans were captured in the same environment, under controlled light conditions; however, these conditions are very different from those of the oral cavity, and the literature must certainly investigate in more detail how much this can affect the quality of the scans [36]. Finally, the SB plays a fundamental role, being the device for transferring the implant position [37–41]. Manufacturing tolerances [37] can cause errors in the transfer of the implant position in the space. This is particularly true for implants with a conical connection, where a minimal tolerance can have important effects on the vertical position of the fixture in the space (i.e. z-axis) with respect to the library. Assembly errors (in the case of SBs composed of two assembled portions), as well as an incongruous screwing [38], can represent other sources of error. Finally, the shape and material of the SBs are important because they respectively influence the behaviour of the CAD superposition algorithm [27, 39] and the absorption or reflection of light [39, 40].

Finally, the present study has some limitations. First, it is an in vitro study. Although scrupulously conducted, and although it is not possible to determine the trueness of an IOS in vivo, an in vitro study cannot exactly reproduce the characteristics present in the patient’s mouth (conditions of light, humidity, saliva). The scanning of plaster models is certainly easier than an intraoral scan, which has limits of space. Furthermore, the patient’s tissues are radically different from a plaster model and have different optical behaviour when hit by light. This must always be kept in mind, although in the edentulous patient to be rehabilitated with FA, no teeth are present. A further limitation of the present work, as already described, lies in the choice of the scanning technique [14], which could have favoured some scanners over others. The use of a desktop scanner for the capture of the reference model could also be considered a limitation. Although this machine is certified for an accuracy of 5 μm, and although this approach has been used in many previous studies [14], a CMM or articulated arm can be considered more reliable tools in capturing reference measurements. Only the centroids at the base (and not the centroids at the top of the SBs) were used for the evaluation of linear and cross distances. Finally, the present study could have collected further and interesting data relating to the linear error increase during scan progression, if only the scan strategy had foreseen the departure from a specific sector of the physical model (x example, right posterior sector only). In fact, as previously reported [5, 16, 17, 25] and recently confirmed by Walter Renne and colleagues in an in vitro study on a dentate model [42], the progression of the scan tends to bring with it an increase in percentage linear error. Unfortunately, this evaluation was not possible in the present study, since the operator was free to start from the right or left posterior area of the model indifferently; the data thus collected do not allow an evaluation of the exact percentage growth of the error along the progression of the scan. In this study, all scans were captured in a specific period (January–February 2020) and therefore with the latest version of the acquisition software available for each of the machines at that time. However, the release of new acquisition software is known to be able to significantly improve the accuracy of an IOS; therefore, the results presented in this study are valid for that period and specific acquisition software. Further studies on the same IOSs with the latest acquisition software are thus needed, to better understand the trueness of the different scanners that are now available.

## Conclusions

The present in vitro study investigated the trueness of 12 IOSs in FA implant impression using three different methods: a mesh/mesh method and a nurbs/nurbs method for the evaluation of the overall trueness, and measurement of linear and cross distances between SBs for the evaluation of local trueness. Statistically significant differences emerged in accuracy between different IOSs, and some may be more suitable for optical impression for the manufacture of implant-supported long-span restorations such as FAs. The results of the overall trueness assessment were confirmed by the local analysis of the distances between the different SBs. Despite some limitations, this study can provide important information relating to the intrinsic error with different IOSs, and therefore useful indications for choosing the ideal machine for FA impression. However, it is important to remember that other factors are important in determining the reliability of an optical impression, including the operator, patient, environmental conditions and SB. Further studies are therefore necessary to understand the weight of each factor in determining the final error in the optical impression.

## Data Availability

The. STL files and the 3D surface models obtained in this study with the different 12 IOS as well as the reference files obtained with the desktop scanner belong to the authors, and are therefore available only upon reasonable request, after approval by all the authors.

## Abbreviations

IOS: Intraoral scanner
3D: Three-dimensional
STL: Standard tessellation language
CAD/CAM: Computer-aided-design/ computer-assisted manufacturing
SC: Single crown
PP: Partial prosthesis
FA: Full-arch
CMM: Coordinate measuring machine
SB: Scanbodies
CAD: Computer-aided-design
NURBS: Non-uniform rational b-spline
PEEK: Polyether-ether-ketone
LED: Light-emitting-diode
SS: Stable scan stage
RM: Reference model
RICP: Robust-iterative-closest-point
PTP: Point-to-plane
SD: Standard deviation
CI: Confidence interval
